# β_1_- and α_2c_-adrenoreceptor variants as predictors of clinical aspects of dilated cardiomyopathy in people of African ancestry

**Published:** 2008

**Authors:** Angela J Woodiwiss, Danelle Badenhorst, Richard Brooksbank, Pinhas Sareli, Gavin R Norton, Karen Sliwa, Rafique Essop

**Affiliations:** Cardiovascular Pathophysiology and Genomics Research Unit, School of Physiology, Division of Cardiology, Chris-Hani Baragwanath Hospital and University of the Witwatersrand, Johannesburg; Cardiovascular Pathophysiology and Genomics Research Unit, School of Physiology, Division of Cardiology, Chris-Hani Baragwanath Hospital and University of the Witwatersrand, Johannesburg; Cardiovascular Pathophysiology and Genomics Research Unit, School of Physiology, Division of Cardiology, Chris-Hani Baragwanath Hospital and University of the Witwatersrand, Johannesburg; Cardiovascular Pathophysiology and Genomics Research Unit, School of Physiology, Division of Cardiology, Chris-Hani Baragwanath Hospital and University of the Witwatersrand, Johannesburg; Cardiovascular Pathophysiology and Genomics Research Unit, School of Physiology, Division of Cardiology, Chris-Hani Baragwanath Hospital and University of the Witwatersrand, Johannesburg; School of Medicine, Division of Cardiology, Chris-Hani Baragwanath Hospital and University of the Witwatersrand, Johannesburg; School of Medicine, Division of Cardiology, Chris-Hani Baragwanath Hospital and University of the Witwatersrand, Johannesburg

## Abstract

**Background:**

Although the β,_1_-adrenoreceptor (AR) Gly389Arg and α_2C_-AR Del322-325 gene variants are associated with the response to β-AR-blocker therapy, whether this effect is associated with the risk for heart failure, or the severity or progression of heart failure is uncertain.

**Aims:**

To assess the relationship between Gly389Arg and Del322-325 variants and the presence, severity and progression of idiopathic dilated cardiomyopathy (IDC) in 403 black South African patients.

**Methods:**

Genotypes were identified using a restriction fragment length polymorphism-based technique and automated sequencing. Left ventricular ejection fraction (LVEF) and dimensions were determined at baseline and in 132 patients after six months of standard medical therapy excluding β-AR-blockers (not indicated as standard care at the time of completing this study).

**Results:**

All patients and controls genotyped for the α_2C_-AR variant were homozygous for the Del322-325 (risk) allele. The Gly389Arg polymorphism was not associated with IDC (control *n* = 429) (Arg389 allele homozygosity: odds ratio = 1.03, confidence limits = 0.78−1.35), nor did it predict LVEF and cavity dimensions either before or after therapy.

**Conclusion:**

In patients homozygous for the risk allele of the α_2c_-AR variant, the β_1_-AR variant neither increased the risk for IDC nor predicted its severity or progression in patients not receiving β-AR-blockers.

## Summary

Persistent β-adrenoreceptor (β-AR) activation promotes progressive heart failure,^1^,^2^ and a cornerstone of therapy in heart failure is the use of blockers of β-ARs.^3^-^10^ There is substantial evidence to implicate the β_1_-AR subtype in the pathogenesis of progressive heart failure.^11^ The activity of β_1_-AR is nevertheless determined in part by functional variants within the gene encoding the receptor. A common polymorphism of the β_1_-AR gene – a substitution of glycine (β_1_Gly389) for arginine (β_1_Arg389) at amino acid 389 – occurs within a Gs-coupling domain.^12^ The β-ARs with the β_1_Arg389 variant have a much greater ability to couple to adenyl cyclase than do those receptors with the β_1_Gly389 variant.^12^ The increased ability of the β_1_Arg389 receptor polymorphism to activate adenyl cyclase may therefore determine the natural history of progressive heart failure or its response to β-AR-blocker therapy.

Although the response to β-AR-blockers in patients with heart failure depends on the position of the 389 genotype of the β_1_-AR gene,^13^-^15^ whether this effect can be attributed in part to an increased risk for the development of heart failure or its progression is not clear.^14^,^16^-^20^ The β1Arg389 variant, when present with a polymorphic α_2c_-AR gene variant, has been reported to increase the risk for developing heart failure in African-Americans.^17^ The α_2_-ARs operate as presynaptic inhibitory receptors that control the release of norepinephrine and influence the progression of heart failure.^21^ A common coding polymorphism of the gene for the α_2C_-AR – the deletion of four consecutive amino acids (Del322-325) – results in a substantial loss of agonist-mediated receptor function in transfected cells.^22^ Since α_2C_-AR activation inhibits norepinephrine release,^23^,^24^ the presence of the α_2C_-AR Del322-325 polymorphism may result in enhanced norepinephrine release and hence increase the risk for heart failure.^25^

Although it appears that the β_1_-AR variant, when present with a polymorphic α_2C_-AR gene variant, increased the risk for heart failure in a group of African-Americans, this relationship was identified in a small study sample (*n* = 78 cases and *n* = 84 controls).^17^ Moreover, in groups of patients of European (*n* = 260 cases and *n* = 230 controls) or Japanese (*n* = 91 cases and *n* = 119 controls) descent, an interaction between these two gene variants and the risk of heart failure has not been confirmed.^26^,^27^ In addition, the impact of these two gene variants on the progression of heart failure in the absence of β-AR-blocker therapy has not been investigated. Therefore, whether the relationship between the position of the 389 β1-AR gene variant and the response to β-AR-blockers in patients with heart failure^13^-^15^ is in part attributed to a relationship between genotype and an increased risk for the development, severity or progression of heart failure is still uncertain.

The aims of the present study were therefore to assess the relationship between the β_1_-AR Gly389Arg and α_2C_-AR Del322-325 variants and (1) the presence, (2) the severity and (3) the progression of idiopathic dilated cardiomyopathy (IDC) in a relatively large sample of black South Africans studied at a time when β-AR-blocker therapy was not indicated as standard care in South Africa.

## Methods

This study was approved by the Committee for Research on Human Subjects of the University of the Witwatersrand (approval number: M951122) and conforms to the principles outlined in the Declaration of Helsinki. The clinical component of the study was conducted between 1995 and 2001 when the use of β-AR-blockers was not considered standard therapy for heart failure in South Africa. All patients gave written informed consent before study entry.

To assess the relationship between the β_1_-AR and α_2C_-AR gene polymorphisms and IDC in this population, a case-control study was performed in which 403 consecutive patients with IDC and 429 age-matched control subjects of the same ethnic origins (African ancestry) were recruited.

Subjects in the control group had a greater body mass index and consisted of more females (Table 1). Patients were recruited if they were ≥ 18 and ≤ 70 years of age, in stable New York Heart Association (NYHA) functional class I to IV heart failure of unknown aetiology, had a left ventricular ejection fraction (LVEF) < 40% as determined by radionuclide ventriculography, and had high-quality echocardiographic images with an LV end-diastolic diameter (LVEDD) > 5.5 cm. Exclusion criteria included evidence of another cause of heart failure and the presence of arrhythmias that could alter LVEF.

**Table 1:** Demographic And Clinical Characteristics Of Patients With IDC And Controls

	*Control (n = 429)*	*IDC (n = 403) All*	*IDC (n = 132) Prospective analysis*
Age (years)	51.4 ± 0.4	51.8 ± 0.8	52.6 ± 1.0
Gender [male/female (%)]	164/265 (62)	199/204 (51)*	83/49 (38)*
Body mass index (kg.m^-2^)	28.1 ± 0.3	25.1 ± 0.4*	25.1 ± 0.5*
Systolic BP (mmHg)	128 ± 1	120 ± 1*	122 ± 2*
Diastolic BP (mmHg)	78 ± 1	78 ± 1	81 ± 1

BP, blood pressure; *p < 0.05 versus controls.

After initial presentation, and following a diagnosis by clinical examination and echocardiography (screening visit), 176 of the 403 patients agreed to participate in a prospective study assessing the impact of the β-AR gene polymorphisms on LV dimensions and function. During the six-month follow up, 24 patients died and 20 were lost to follow up. Of the remaining 132 patients who were followed prospectively, 71 were newly diagnosed. The demographic and clinical characteristics of the subgroup of IDC patients on whom follow-up LV structure and function was assessed were comparable in their demography and clinical characteristics to those of the total group of IDC patients assessed (Table 1).

These 132 patients who were followed prospectively received treatment with digoxin and diuretics (furosemide) for seven days and then angiotensin-converting enzyme inhibitors (ACEI) were added to their therapy. These patients were followed for six months. Monthly visits were scheduled for clinical assessment and evaluation of the patient’s adherence to therapeutic agents.

Clinical examinations, echocardiographic assessments and radionuclide studies were performed at baseline, and then repeated at six months. The primary endpoints were LVEF determined using radionuclide ventriculography, and LVEDD determined using echocardiography. Radionuclide ventriculography as opposed to echocardiography was used as the method of preference to assess the impact of β_2_-AR genotype on LV systolic function, as this measurement is not subject to observer bias. To show a 10-point difference in radionuclide LVEF between groups with 80% power after six months of therapy required a sample size in each group of 21 patients.

## Functional class, echocardiography and radionuclide studies

A physician assessed the NYHA functional class of the patients during the baseline and follow-up visits. The same physician evaluated all patients. A multiple-gated equilibrium cardiac blood pool scintigraphic technique was used to measure LVEF (Elscint Apex 409).^28^ Imaging was performed in the left anterior oblique projection providing the best septal separation of the ventricles with a 0−10° caudal angulation. Calculations of LV performance were made as previously described,^29^ using an automatic edge-detection algorithm for the determination of LV borders. A single observer interpreted all studies.

Two-dimensional targeted M-mode echocardiography with doppler colour-flow mapping was performed using a Hewlett Packard Sonos 5500 echocardiograph attached to a 2.5 or 3.5 MHz transducer. All studies were performed and interpreted by the same operator and recorded on videotape. Left ventricular dimensions were measured according to the American Society of Echocardiography guidelines.^30^ Measurements of LV dimensions and function were determined on an average of ≥ three beats. The investigators that performed and interpreted the radionuclide and echocardiographic studies were unaware of the treatment assigned to patients.

## Genotyping

Blood for genetic studies was obtained during the initial screening period. Deoxyribonucleic acid (DNA) was extracted from whole blood using standard techniques, as previously described.^31^ Genotyping, undertaken after the clinical component of the study was complete, was performed by an investigator (DB) unaware of the identity of the patients from whom DNA was obtained.

Genotyping of the Gly389Arg variant of the β_1_-AR gene was undertaken using a polymerase chain reaction (PCR)-restriction fragment length polymorphism-based technique employing the appropriate primer pairs and restriction enzymes. DNA was amplified using 5′-CGCTCTGCTGGCTGCCCTTCTTCC-3′ and 5′-TGGGCTTCGAGTTCACCTGCTATC-3′ forward and reverse primers respectively. PCR was carried out in a total volume of 20 μl containing ~ 50 ng DNA, 1 × PCR buffer (Takara), 2 mM MgCl_2_, 0.2 mM dNTP, 2.5 mM forward and reverse primers, 6% dimethylsulfoxide, 1 μg.ml^-1^ bovine serum albumin, and 1 unit Taq polymerase (Takara).

The PCR conditions were as follows: 94°C for four minutes, followed by 30 cycles of denaturation (94°C for one minute per cycle), annealing (60°C for 45 seconds) and extension (72°C for one minute) with a final extension step at 72°C for four minutes. The Arg389 allele PCR product contains a unique site for restriction by 1 333 units of Bcg*I* (three hours at 37°C). Cleavage of the 530-bp product into 342- and 154-bp fragments confirms the presence of this allele. BcgI (New England Biolabs) cleaves twice to excise its recognition site, accounting for the 34-bp discrepancy in the fragments generated. The restriction digests were electrophoresed on 3% agarose gels and visualised with ethidium bromide staining and ultraviolet illumination. To avoid misgenotying as a consequence of failure of restriction enzyme digestion, a known heterozygous sample for each of the polymorphisms was included in each PCR, digestion procedure and gel, and all samples were genotyped in duplicate.

Genotyping of the Del322-325 variant of the α_2c_-AR gene was undertaken using automated sequence analysis of PCR fragments.^32^ DNA was amplified using 5′-AGCCCGACGAGAGC-AGCGCA-3′ and 5′-AGGCCTCGCGGCAGATGCCGTACA-3′ forward and reverse primers respectively. PCR was carried out in a total volume of 20 μl containing ~ 50 ng DNA, 1 × PCR buffer (Takara), 2 mM MgCl_2_, 0.2 mM dNTP, 2.5 mM forward and reverse primers, 12% dimethylsulfoxide and 1 unit Taq polymerase (Takara).

The PCR conditions were as follows: 94°C for four minutes, followed by 40 cycles of denaturation (94°C for 40 seconds per cycle), annealing (59°C for 30 seconds per cycle) and extension (72°C for 30 seconds per cycle) with a final extension step at 72°C for seven minutes. PCR products were purified using shrimp alkaline phosphatase and *E coli* exonuclease I (Fermentas). Samples were processed using the BigDye version 3.1 Dye Terminator Cycle Sequencing Kit (Applied Biosystems) according to the manufacturer’s instructions on the Genetic analysis system SCE2410 (SpectruMedix LLC, Pennsylvania, USA).

Analysis was performed using BaseSpectrum V2.1.1 software (SpectruMedix LLC, Pennsylvania, USA). All patients (*n* = 50) and controls (*n* = 50) genotyped for the Del322-325 variant of the α_2c_-AR gene were homozygous for the Del322-325 allele. Hence not all patients or controls were genotyped for the Del322-325 variant of the α_2c_-AR gene.

## Analyses

Data are presented as mean ± SEM. Case and control group mean values were compared with the use of a two-sample Student’s *t*-test or a Mann-Whitney test [depending on whether variables were nominal or ordinal (Bartlett’s test)]. To test for Hardy-Weinberg equilibrium, the expected genotype numbers were calculated from the allele frequencies and deviation from the observed genotype numbers determined using a χ^2^ test. Effects of alleles on the presence of IDC were evaluated using a χ^2^ test.

Genotype effects on the presence of IDC were assessed with logistic regression analysis with age, gender and body mass index (BMI) included as covariates. To assess the effect of genotype on either LVEF, or cardiac dimensions, a MANCOVA was performed with age, gender, disease duration and BMI included in the regression model. A paired Student’s *t*-test was used to detect changes from baseline. Analysis of covariance adjusting for baseline data, age, gender, disease duration and type of ACEI was employed to determine differences in changes in LV cavity size and function, and final LV cavity size and function between genotype groups. Genotype effects on final LV cavity size and function were assessed using MANCOVA with age, gender, BMI, disease duration and baseline data included as covariates.

## Results

No significant differences were noted in the demographic or general clinical data in genotype-specific subgroups of patients on whom LV structure and function was determined (Table 2).

**Table 2:** Baseline Demographic And Clinical Characteristics Of Patients With IDC Prospectively Studied, Grouped According To β_1_-Adrenoreceptor Genotype

*Gene variant*	*Gly389Arg*		
*Genotype group*	*Arg389 (CC) (n = 70)*	*Gly389Arg (GC) (n = 47)*	*Gly389 (GG) (n = 15)*
Age (years)	52.7 ± 1.4	50.8 ± 1.7	52.9 ± 2.7
Gender [male/female (%)]	44/26 (37)	27/20 (43)	12/3 (20)
Body mass index (kg.m^-2^)	25.2 ± 0.5	25.2 ± 0.7	25.0 ± 1.2
Systolic BP (mmHg)	121 ± 2	123 ± 3	123 ± 5
Diastolic BP (mmHg)	81 ± 2	80 ± 2	81 ± 3
Functional class (I/II/III/IV)	2/32/34/2	1/23/22/1	0/8/7/0
Disease duration (months)	12.1 ± 2.2	12.2 ± 3.1	9.3 ± 4.9
Perindopril/enalapril/trandolapril (%)	40/46/14	34/43/23	53/20/27

## α_2c_-AR genotype and IDC

All cases and controls genotyped for the Del322-325 variant of the α_2c_-AR gene were homozygous for this variant.

## Association between β_1_-AR genotype and IDC

Both the case and the control groups were estimated to be in Hardy-Weinberg equilibrium. The χ^2^ values when comparing expected and actual genotype numbers were 2.72 and 1.84 for the case and the control groups respectively (Table 3). No differences were noted in the allele frequencies between case and control groups (Table 3). The β_1_-AR genotype was not an independent predictor of IDC (logistic regression: CC vs GG, β-coefficient = −0.07 ± 0.32, *p* = 0.83; CC vs GC, β-coefficient = −0.11 ± 0.21, *p* = 0.60; CC vs GC + GG, β-coefficient = −0.10 ± 0.19, *p* = 0.61).

**Table 3:** β_1_-Adrenoreceptor Genotype And Allele Frequencies Of Patients With IDC And Controls

	*β_1_-adrenoreceptor gene Gly389Arg polymorphism*				
	*Genotype*			*Allele*	
	*Arg389 (CC)*	*Gly389Arg (GC)*	*Gly389 (GG)*	*Arg389 (C)*	*Gly389 (G)*
Control (*n* = 429)	210 (49)	172 (40)	47 (11)	592 (69)	266 (31)
IDC (*n* = 403)	200 (50)	161 (40)	42 (10)	561 (70)	245 (30)
IDC* (*n* = 132)	70 (53)	47 (36)	15 (11)	187 (71)	77 (29)

Numbers represent sample numbers (%). *Represents patients studied prospectively. No relationship between genotype (logistic regression analysis adjusting for age, gender and body mass index) or allele (χ^2^ analysis) and the presence of IDC was noted (see text for values).

## Association between β_1_-AR genotype and LV function and cavity dimensions in IDC

A similar number of patients in each β_1_-AR genotype-specific group died or were lost to follow up (data not shown). All genotype groups received similar doses and type of drug therapy (type of angiotensin-converting enzyme inhibitor is indicated in Table 2). The Gly389Arg polymorphism of the β_1_-AR gene failed to predict baseline LV function or cavity dimensions (Table 4). Following six months of therapy, LVEF (MUGA) increased by 7.0 ± 1.0 absolute units (*p* < 0.0001), LVEDD decreased by 0.27 ± 0.06 cm (*p* < 0.02), and LV end-systolic diameter decreased by 0.38 ± 0.07 cm (*p* < 0.01) in all patients considered together. The increase in LVEF and decrease in LVEDD and LVESD were the same in β_1_-AR genotype-specific groups (Table 4). At the end of the study, the β_1_-AR genotype failed to predict LV function and cavity dimensions (Table 4).

**Table 4:** Left Ventricular Chamber Dimensions And Function In Patients With IDC Prospectively Studied, Grouped According To β_1_-Adrenoreceptor Genotype

*Gene variant*	*β_1_-Gly389Arg*								
*Genotype group*	*Arg389 (CC) (n = 70)*			*Gly389Arg (GC) (n = 47)*			*Gly389 (GG) (n = 15)*		
	*Baseline*	*Final*	*Change*	*Baseline*	*Final*	*Change*	*Baseline*	*Final*	*Change*
LVEDD (cm)	6.67 ± 0.09	6.35 ± 0.11	−0.28 ± 0.09	6.44 ± 0.12	6.29 ± 0.13	−0.21 ± 0.11	6.33 ± 0.22	5.99 ± 0.28	−0.39 ± 0.15
LVESD (cm)	5.84 ± 0.09	5.41 ± 0.13	−0.39 ± 0.10	5.66 ± 0.12	5.40 ± 0.15	−0.34 ± 0.13	5.43 ± 0.22	4.98 ± 0.34	−0.50 ± 0.16
LVEF (%)	23.5 ± 0.9	30.9 ± 1.5	7.2 ± 1.5	24.2 ± 1.00	30.3 ± 1.8	6.1 ± 1.4	24.6 ± 2.5	34.0 ± 3.9	9.4 ± 2.8

LVEDD, left ventricular end-diastolic diameter; LVESD, left ventricular end-systolic diameter; LVEF, left ventricular ejection fraction.

## Discussion

The main findings of the present study are as follows: a common coding polymorphism of the gene for the α_2C_-AR – a deletion of four consecutive amino acids (Del322-325) – that results in a substantial loss of agonist-mediated receptor function in transfected cells^22^ appears to be ubiquitous in black South Africans. In this population group, the Gly389Arg polymorphism of the β_1_-AR gene is not associated with IDC, nor does it predict the degree of systolic dysfunction and dilatation at baseline or progression after six months of standard medical therapy (excluding β-AR blockers) in IDC.

Although a number of studies have indicated that the β_1_-AR gene variant at position 389 either alone or in synergy with the α_2C_-AR Del322-325 variant modifies the response to β-AR-blocker therapy in heart failure,^13^-^15^ whether this change is in part attributed to an impact of these gene variants on the risk for the clinical expression of the phenotype for heart failure, the severity of heart failure, or the progression of heart failure independent of β-AR-blocker therapy is uncertain. The present study provides the first clear evidence to indicate that even on a genetic background of homozygosity for the risk allele of the α_2C_-AR Del322-325 variant, the β_1_-AR gene variant at position 389 does not influence either the risk for the clinical expression of the phenotype for heart failure, the severity of heart failure, or the progression of heart failure independent of β-AR-blocker therapy.

Unlike the apparent consistency of the data indicating that the β_1_-AR gene variant at position 389 modifies the response to β-AR-blocker therapy in heart failure,^13^-^15^ as with many genetic variants studied to date, the reported relationships between the functional Gly389Arg polymorphism of the β_1_-AR gene and heart failure have been inconsistent. Although studies conducted in transgenic mice have demonstrated that the β_1_-Arg389 variant predisposes to a depressed ventricular function and pathological fibrosis,^14^ several studies relating the Gly389Arg polymorphism of the β_1_-AR gene with the risk for human heart failure or IDC and the progression of these diseases have produced inconsistent data.^14^,^16^-^20^

One potential explanation for the inconsistencies in the reported relationships between the Gly389Arg polymorphism of the β_1_-AR gene and heart failure is that an α_2C_-AR gene variant influences the impact of the β_1_-AR gene variant. Indeed, the presence of an α_2C_-AR Del322-325 polymorphism has been shown to increase the chances that the β1Arg389 variant confers an increased risk for heart failure in 79 African-Americans with heart failure and 84 control subjects.^17^ This interactive effect between the β_1_Arg389 and α_2C_-AR Del322-325 polymorphisms has also been examined in a much larger group of individuals of European descent, and in a group of patients of Japanese descent but the interaction was not reproduced.^26^,^27^

The present study is the first to assess the role of the β_1_-AR and the α_2C_-AR gene variants in a relatively large study sample of patients (*n* = 403) and controls (*n* = 429) of African descent. In the present study we were unable to show a relationship between the β_1_Arg389 gene variant and IDC in black South Africans who are homozygous for the risk α_2C_-AR Del322-325 allele. Moreover, in a prospective study we were unable to show a relationship between the β_1_Arg389 variant and either the degree of systolic dysfunction or the extent of cardiac dilatation at baseline or after six months of standard medical therapy, but without β-AR-blockers in black South Africans with IDC who were homozygous for the risk α_2C_-AR Del322-325 allele.

In 79 African-Americans with heart failure and 84 control subjects, homozygosity for the β_1_-Arg389 and α_2C_-AR Del322-325 alleles produced an odds ratio for heart failure of 3.46 (confidence interval 0.68−17.6).^17^ In contrast, in the present study, homozygosity for the β_1_-Arg389 variant in subjects homozygous for the Del322-325 allele produced an odds ratio for heart failure of 1.03 (confidence interval 0.78−1.35). As with many genetic association studies, although studies with small sample sizes, such as that reported on previously,^17^ may show associations with disease phenotypes, there is a reduced chance of showing an effect with increasing sample sizes as in the study presently described. Moreover, in order to maintain consistency, we studied a sample of patients with IDC rather than recruiting patients with mixed forms of heart failure, such as IDC and ischaemic dilated cardiomyopathies, as reported on by Small *et al.*^17^ Whether genetic effects in IDC and ischaemic dilated cardiomyopathy are similar is unknown. Nevertheless, a large study in Caucasian patients with heart failure due to either coronary heart disease or IDC reported no increased risk associated with homozygosity for both the β_1_-Arg389 variant and the Del322-325 allele.

Lastly, it is possible that although groups of patients of African descent were studied by both Small *et al*.^17^ and our group, different environmental and genetic factors may contribute to heart failure in African-Americans and black South Africans. Indeed, differences in allele frequencies were evident between the two groups, which is not surprising given that there is a higher degree of genetic admixture in African-Americans compared to Africans living in Africa.^33^

Although, as the present study indicates, it is unlikely that the β_1_-AR gene variant at position 389 has a pathophysiological role to play in the clinical expression of IDC or its progression independent of β-AR-blockers in groups of African descent, the α_2C_-AR Del322-325 polymorphism may nevertheless still have an important role to play in this ethnic group. Previous studies have demonstrated a substantially increased risk of heart failure in African-Americans with the α_2C_-AR Del322-325 allele.^17^ Moreover, Regitz-Zagrosek *et al.*^34^ have demonstrated an association between the presence of the deletion polymorphism and reduced event and death rates in patients with IDC. In the present study the presence of homozygosity for the α_2C_-AR Del322-325 allele in all patients (*n* = 50) and controls (*n* = 50) genotyped precluded us from studying the impact of this genetic variant in isolation on IDC in this population group.

The limitations of the present study were as follows: first, we did not genotype all cases and controls for the α_2C_-AR Del322-325 polymorphism. However, in the 100 individuals genotyped using sequencing techniques (50 cases and 50 controls), all individuals were homozygous for the Del322-325 allele. Second, as with all case-control studies, we did not account for population stratification. However, the selection of controls was from the same ethnic group and geographic location (Soweto) as the cases, and the study sample was relatively large. Third, prospective follow-up was for only six months. However, this period was selected because beyond six months, mortality and morbidity related to heart failure would have limited the ability to appropriately assess LV structural and functional changes.^35^ Fourth, LV structure and function were only assessed at rest rather than during exercise. It is therefore possible that we could have missed an impact of β_1_-AR genotype on systolic function during exercise. However, the main hypothesis of the present study was related to the risk for IDC and the impact on structure and function at baseline and after six months in the absence of β-AR-blocker therapy, rather than the impact of β_1_-AR genotype on exercising function.

## Conclusion

The present study demonstrates that black South Africans are ubiquitously homozygous for the risk α_2C_-AR Del322-325 variant, and that with this high-risk genetic background for the α_2C_-AR Del322-325 variant,^18^ the Gly389Arg polymorphism of the β_1_-AR gene neither predicts an increased risk for the expression of the clinical phenotype of heart failure in IDC, nor determines disease severity or progression of IDC after six months of standard medical therapy in the absence of β-AR blockers in this population group. These data suggest that the relatively consistent relationship noted between the β_1_-AR Gly389Arg variant and the response to β-AR-blocker therapy and heart failure previously reported on^13^-^15^ is unlikely to be determined by an association between genotype and the risk for the expression of the clinical phenotype of heart failure, the severity of heart failure or the progression of the disease.
